# Influence of the Form of Administration of Chlorogenic Acids on Oxidative Stress Induced by High fat Diet in Rats

**DOI:** 10.1007/s11130-017-0608-3

**Published:** 2017-04-08

**Authors:** G. Budryn, D. Zaczyńska, D. Żyżelewicz, J. Grzelczyk, Z. Zduńczyk, J. Juśkiewicz

**Affiliations:** 1 Institute of Food Technology and Analysis, Faculty of Biotechnology and Food Sciences, Lodz University of Technology, 90-924, Lodz, Poland; 2 Institute of Animal Reproduction and Food Research of Polish Academy of Sciences, 10-748 Olsztyn, Poland

**Keywords:** Green coffee, Chlorogenic acids, β-cyclodextrin, Rat model

## Abstract

Green coffee is one of health-promoting supplements of the diet, applied in the form of either preparations or enriched food products. Its positive impact is manifested by mitigation of the development of certain tumors, *e.g.*, in the colon and liver, and type 2 diabetes. Many studies proved that chlorogenic acids are the main active substances in green coffee. The bioavailability of these compounds depends among others on their interactions with other components of the diet, mainly proteins. When they are used as food ingredients, their bioavailability is additionally decreased because of the decomposition or interactions with other ingredients during food processing. The undesirable changes may be limited among others by microencapsulation, for example with β-cyclodextrin. In this study, rats were fed the pro-oxidative high fat diet, which was supplemented with chlorogenic acids from green coffee that were used in four forms such as: a purified extract, complexes of chlorogenic acids and β-cyclodextrin, and bread supplemented with either the extract or the β-cyclodextrin inclusion complex. Chlorogenic acids added to bread because of the reduced absorption from the crumb in the small intestine and increased passage to the colon, contributed to the beneficial modification of enzymatic activities of intestinal microbiota. When added directly to the diet, they contributed to the improved antioxidant status in the liver and kidneys, lowered glucose level and increased HDL level. A high ratio of reduced to oxidized glutathione in the liver and a high concentration of antioxidants in the blood serum were observed after administration of chlorogenic acids in the form of inclusion complexes with β-cyclodextrin, indicating that microencapsulation increased their bioaccessibility due to the limited interactions with other components of the diet.

## Introduction

Supplementation of diet has been increasingly regarded as a method of many diseases prevention [1, 2]. Certain health-promoting properties of food are ascribed to phenolic substances, including phenolic acids of benzoic and hydroxycinnamic families [3]. Coffee beans are a rich source of the latter acids [4]. Green coffee contains caffeic and ferulic acids in the form of mono- and diesters with quinic acid, referred to as chlorogenic acids (CGAs) [5, 6]. CGAs exhibit a broad range of biological activities: anti-thrombotic, anti-inflammatory, hypoglycemic and antioxidant [7–10]. CGAs decrease the risk of several oxidative stress-related diseases, including atherosclerosis, some kinds of cancer and type 2 diabetes [11–13]. Because the concentration of CGAs is significantly reduced by roasting, these compounds should be extracted from green coffee. To study the influence of CGAs-enriched food on human health, green coffee extract was added to bread, which is one of commonly consumed products [14]. However, during food processing and storage, polyphenols, including CGAs, may undergo many conversions that can affect their bioavailability and health-promoting effects. The conversions include the non-enzymatic and enzymatic oxidation, polymerization, non-covalent and covalent interactions with proteinaceous substances, incorporation into high molecular mass substances, like melanoidins, and others [3]. The polyphenols added to foods as health promoting ingredients may be protected by microencapsulation using yeast cells, liposomes, starch or β-cyclodextrin. Polyphenols contained in the core of the microcapsules are stabilized during food storage and processing, and are bioavailable after digestion of the shell that is observed already in the upper gastrointestinal tract [15, 16]. The objective of this study was to evaluate the health promoting activity of CGAs, manifested as reduction of oxidative stress in rats that were fed the pro-oxidative high fat diet, supplemented with either free or complexed CGAs, or bread enriched with either free or complexed CGAs.

## Materials and Methods

### Chemicals and Raw Materials

Analytical-grade ethanol and ethyl acetate were purchased from POCh (Gliwice, Poland). Ultrapure water (the resistivity of 18.2 MΩ cm) was obtained from a Millipore Milli-Q Plus purification system (Bedford, MA, USA). Green Robusta coffee beans (*Coffea canephora* L.) harvested in Brazil in 2014, and dehulled by the dry method, were purchased from Bero Polska (Gdynia, Poland).

### Obtaining and Purification of Green Coffee Extract (GCE)

An aqueous extract from grounded green coffee beans was obtained by incubation at 110 °C for 10 min [17]. The solution was freeze-dried in a DELTA 1-24LSC Christ drier (Osterode am Harz, Germany) and purified by centrifugal partition chromatography using a SPOT Prep II 50 chromatograph (Armen Instrument, Saint-Avé, France) integrated with a UV/VIS detector and a fraction collector. The two-phase system of solvents contained water, ethanol and ethyl acetate (5:1:4, *v*/v/v). Elution of CGAs was monitored by absorbance measurements at 320 nm. The fractions rich in CGAs were combined and the extract was concentrated using a ScanMaxiVac Labogene concentrator (Lynge, Denmark) and freeze-dried again. The profile of CGAs in the purified extract was analyzed by LC-MS as previously described [18]. The concentration of CGAs was 54.35 g/100 g of the dried purified extract. The extract was also analyzed for the concentrations of soluble dietary fiber (AOAC 991.19), protein (AOAC 2001.11), ash (AOAC 942.05), and water (AOAC 934.01). It contained 9.28 g/100 g of carbohydrates, 2.98 g/100 g of protein, 12.16 g/100 g of dietary fiber, 19.13 g/100 g of ash and 2.13 g/100 g of water.

### Microencapsulation of CGAs with β-Cyclodextrin (β-CD)

Inclusion complexes of β-CD and CGAs (βCD-CGAs) were obtained using an excess of polyphenols to maximize the efficacy of complexation. Portions of β-CD (11.35 g) and GCE (13.03 g containing 7.08 g of CGAs) were dissolved in 200 mL of water, incubated for 2 h at 50 °C with continuous stirring, and left for 24 h at 0 °C. The suspension formed was centrifuged using a MIKRO 22R Hettich centrifuge (Kirchlengern, Germany) at 4 °C for 20 min at 10000 x g. The precipitate containing a mixture of β-CD and CGAs complexes was characterized by LC-ESI-MS/MS as described in the previous work [18]. The washed and freeze-dried precipitate contained 20.50 g/100 g of CGAs, 78.42 g/100 g of carbohydrates (β-CD) and 1.08 g/100 g of water.

### Supplemented Bread Preparing

The ingredients used in formulation of bread are presented in Table 1. A half of water dose was mixed with yeast, sugar and ¼ of the flour dose. The remaining ingredients, including CGAs preparations, were added after 20 min pre-fermentation at 33 °C. The concentration of CGAs preparations in the bread was equivalent to 0.5% CGAs in the diet. The dough was left for 30 min for further fermentation. After that the loaf was formed and baked in a Piccolo PRO II Winkler Wachtel (Düsseldorf, Germany) baking oven at 200 °C for 55 min [4].

**Table 1:** Formulation of bread supplemented with CGAs (g/100 g of the final product)

Ingredient	CB	BGCE	BβCD-CGAs
Wheat flour	50.0	48.7	47.1
Yeast	5.0	5.0	5.0
Sugar	1.0	1.0	1.0
Salt	0.7	0.7	0.7
Water	43.3	43.3	43.3
Green coffee extract	-	1.3	-
β-CD-CGAs inclusion complex	-	-	2.9

*CGAs*–Chlorogenic acids; *CB* Control bread without additives; *BGCE* Bread supplemented with chlorogenic acids in the form of green coffee extract; *BβCD-CGAs* Bread supplemented with chlorogenic acids in the form of inclusion complexes with β-cyclodextrin

### Animal Study

The study was conducted according to the protocol approved by the Local Institutional Animal Care and Use Committee (permission no. 71/2014; Olsztyn, Poland) on selected Wistar rats of similar age and body weight. Experimental groups consisted of eight male rats. The basic diet was a semi-synthetic modification [19] of an AIN-G93G diet developed by the American Institute of Nutrition. The diets C (standard) and CF (pro-oxidative - high-fat) were regarded as controls. The C diet provided sufficient amounts of dietary fiber (5% of cellulose), right share of energy from fat (7% of rapeseed oil) and highly digestible carbohydrates (10% of sucrose and 53% of corn starch). This diet contained also 0.3% of DL-methionine, 0.2% of choline chloride, 3.5% of mineral mixture and 1% of vitamin mixture. The CF diet was the modification of the C diet, adapted for the ongoing experiment to verify the positive effect of CGAs on selected physiological indices in the oxidatively stressed rats. Palm oil with a high ratio of n-6/n-3 acids (>122:1) was used as a pro-oxidative factor. The CF diet contained more palm oil (14%) and less corn starch (11%) and cellulose (3%). The pro-oxidative diets supplemented with CGAs in the form of either GCE (FGCE) or βCD-CGAs complexes (FβCD-CGAs) contained 0.5% of CGAs that corresponded to 0.95% of GCE and 2.10% of βCD-CGAs, respectively. These preparations replaced a part of corn starch. Energetic values of the experimental C and CF diets were estimated according to the Research Diets, Inc. (New Brunswick, NJ, USA). When the diet of rats was based on bread, the control diet with bread (CB) consisted of 73% of bread, 7.8% of casein, 0.2% of DL-methionine, 14% of palm oil, 0.2% of choline chloride, 0.3% of cholesterol, 3.5% of mineral mix, and 1% of vitamin mixture. The modified diet contained bread supplemented with CGAs in the form of either GCE or βCD-CGAs (the BGCE or BβCD-CGAs diets, respectively). The diets were administered during the period of four weeks, with everyday control of feed intake. The rats were used in compliance with the European guidelines for the care and use of laboratory animals. The animals were maintained individually in metal cages at a stable temperature (21–22 °C), a 12 h light/12 h dark cycle and a ventilation rate of 15 air changes per hour. During the whole nutritional test, samples of faeces were collected for analyses. After four weeks of experimental feeding, the rats were weighed and anesthetized with sodium pentobarbital (50 mg/kg body weight) [19]. Biochemical analyses of blood serum, cecum and colon were performed post mortem. Blood was collected into tubes containing heparin, and then centrifuged at 8000 x g for 15 min, frozen in liquid nitrogen and stored at −70 °C. Blood biochemical markers were assayed using a biochemical analyzer Pentra 200 (Horiba Medical, Montpellier, France) and appropriate reagent kits provided by the manufacturer. The dissected colon and cecum and their contents were weighed and pH of the contents was measured using microelectrodes (a 301 pH-meter, Hanna Instruments, Portugal). The short chain fatty acids (SCFA) concentration in the cecum contents was determined using a gas chromatograph (GC-14A Shimadzu, Kyoto, Japan) equipped with a glass column (2.5 × 2.6 mm) containing 10% of SP-1200/1% H3PO4 on 80/100 Chromosorb AW. The temperatures of the column, FID detector and injection were 110, 180 and 195 °C, respectively. Portions of cecum contents (0.2 g) were mixed with formic acid, diluted with deionized water, and centrifuged 12,000 x g for 5 min. The supernatant was applied to the chromatographic column [19]. The activities of β-glycolytic enzymes in faeces: β-glucuronidase, β-galactosidase and β-glucosidase were determined by measurements of the amount of *p*- or *o*-nitrophenol released from the substrates [19]. Before the assays, samples of faeces were dissolved in 0.1 M phosphate buffer (pH 7) and centrifuged at 10000 x g. The reaction mixtures contained 0.3 ml of substrate solution (5 mM in 0.1 M phosphate buffer pH 7) and 0.2 ml of faeces solution (*v*/v, 1:10). After 10 min incubation at 37 °C the reaction was stopped by adding 2.5 ml of 0.25 M sodium carbonate. The concentrations of *p*- and and *o*-nitrophenol were measured at 400 and 420 nm, respectively. The enzymatic activity (U) was expressed in μmol of *p*-(*o*-)nitrophenol released in 1 h by 1 g of faeces. The antioxidant capacities of plasma water and lipid fractions (ACW and ACL, respectively) were determined using a Photochem® device (Analytik Jena, Leipzig, Germany) and analytical kits provided by the manufacturer for assays of the antioxidant activity of hydrophilic and lipophilic compounds. Measurements of fat and lean mass within the body of rats were performed intravitally by the nuclear magnetic resonance technique, using a Minispec LF90 II device (Bruker Optics, Germany). The concentration of glutathione in its oxidized and reduced form (GSH and GSSG, respectively) in the liver was determined by the enzymatic recycling method and expressed in μmol GSH or GSSG per g of tissue [19]. The contents of substances reacting with thiobarbituric acid (TBARS) in kidneys, liver and heart were determined spectrophotometrically at 532 nm and expressed in ng of malondialdehyde per g of tissue [19].

### Statistical Analysis

**T**he type of diet was a differentiating factor, and one-way analysis of variance ANOVA was applied using Statistica 10.0 software. The SEM - summed standard error was calculated as the sum of standard errors for all rats divided by the square root of the total number of rats. The significance of differences between the groups of rats was determined at a confidence level of *P* ≤ 0.05 [19].

## Results and Discussion

### Body Weight, Diet Intake, Body Composition and Indices of the Cecum and Colon in Rats

The diets tested influenced the intake of feed and body weight gain in experimental rats (Table 2). The groups fed the FGCE and FβCD-CGAs diets were characterized by a slower weight gain than expected after administration of the pro-oxidative high fat diet. Feeding the CB, BGCE and BβCD-CGAs diets resulted in a similar weight gain in rats (*P* > 0.05). Contrary to many reports describing the body weight reduction caused by the regular consumption of roasted and green coffee [20], in our study this effect occurred only at an excessive intake of fat (the CF, FGCE and FβCD-CGAs diets). This suggests that caffeine could play the crucial role in weight reduction with proper diet (no significant effects with CB, BGCE and BβCD-CGAs diets, *P* > 0.05) [21]. The GCE contained some fiber, which may reduce the bioavailability of fats and can also lead to a limited weight gain [22]. No effect of coffee formulations on weight gain was observed by Rao et al. [23], who considered the fact as beneficial for the potential use of coffee as anticancer drug because of the lack of cachexia symptoms. The similar trend was observed when the fat mass was compared. The significant decrease in the body fat mass and increase in lean mass was observed in the BβCD-CGAs diet group compared to the other groups (*P* ≤ 0.05). The group fed the BβCD-CGAs diet was characterized by a greater mass of cecum wall and the substantially greater filling with contents compared to the other groups of rats (*P* ≤ 0.05). There were no significant differences in hydration of cecum contents between the groups (*P* > 0.05). In the BβCD-CGAs diet group, the lower pH and lower concentration of ammonia in the cecum and colon contents suggested the decreased activity of urease-secreting pathogenic and putrefactive bacteria, and the increased activity of bifidogenic ones.

**Table 2:** Body weight, diet intake, body composition and indices of the cecum and colon in rats

Index	C	CF	FGCE	FβCD-CGAs	CB	BGCE	BβCD-CGAs	SEM	*P*
Initial body weight (g)	280^a^	280^a^	280^a^	280^a^	281^a^	281^a^	281^a^	1.301	0.091
Final body weight (g)	364^a^	392	379	384	365^a^	365^a^	364^a^	2.089	0.076
Body weight gain (g)	84^a^	112	99	104	84^a^	84^a^	83^a^	1.626	0.065
Diet intake (g/day)	19.7^d^	19.2^b^	19.6^c,d^	18.7^a^	19.3^b^	19.4^b,c^	18.6^a^	0.144	0.082
Fat mass (g)	123.8^a^	148.5	132.0	145.3	123.6	123.3^a^	115.3	1.536	0.180
Lean mass (g)	128.0	125.2	126.0	127.3	128.7	129.2	140.8	1.732	0.134
Cecum:									
Tissue^1^	0.254^a^	0.199	0.272^b^	0.257^a^	0.276^b^	0.299	0.359	0.009	0.103
Contents^1^	0.460	0.403	0.484	0.572	0.502	0.725	1.010	0.051	0.201
NH_3_ (mg/g)	0.320	0.339	0.285^a^	0.298^b^	0.299^b^	0.284^a^	0.275	0.007	0.125
pH of contents	7.25	7.26	7.14	7.26	7.23	6.97	6.93	0.036	0.208
Colon:									
Tissue^1^	0.267^a,b^	0.264^a^	0.271^b^	0.299^c^	0.295^c^	0.272^b^	0.284	0.005	0.199
Contents^1^	0.190^a^	0.184	0.190^a^	0.207	0.197	0.218	0.235	0.013	0.287
pH of contents	6.85^b^	6.83^b^	6.59^a^	6.57^a^	6.83^b^	6.68	6.49	0.048	0.019

*C* Control diet; *CF* Control high fat diet; *FGCE* High fat diet supplemented with chlorogenic acids in the form of green coffee extract; *FβCD-CGAs* High fat diet supplemented with chlorogenic acids in the form of inclusion complexes with β-cyclodextrin; *CB* Control diet with bread without additives; *BGCE* Diet with bread supplemented with chlorogenic acids in the form of green coffee extract; *BβCD-CGAs* Diet with bread supplemented with chlorogenic acids in the form of inclusion complexes with β-cyclodextrin; *SEM* Summed standard error; *n* = 56; ^1^ g/100 g of body weight (BW); the same letter in one line means no significant differences (*P* > 0.05)

### The Activity of Bacterial Enzymes in the Faeces of Rats during the Experiment

The applied nutritional treatments differentially affected the activity of bacterial enzymes in rats’ faeces (Table 3). The decline in β-glucosidase activity was observed in all groups. The decrease was particularly apparent in the groups consuming bread supplemented with both forms of CGAs. The greatest increase of microbial β-galactosidase activity on the 4th day of the experiment was observed in the rats fed the diet CF while the administration of the FGCE and FβCD-CGAs diets caused the lesser increase of this activity, which was decreased in the other groups. On the next days, the activity of β-galactosidase was decreasing in all groups. The analysis of enzymatic activities showed that on 11th day the highest decrease in β-galactosidase activity was observed in the groups, for which the CGAs-supplemented bread was the main component of the diets. This activity was the lowest in the BβCD-CGAs diet group (*P* < 0.05). The latter diet led also to the strong decrease in bacterial β-glucuronidase activity. The latter activity was lower in the FGCE and FβCD-CGAs diet groups than in the CF diet group. It was the lowest in the C diet group, which did not contain the excess of fat. β-glycosidases are synthesized among others by pathogens such as *Bacteroides, Enterocuccus, Escherichia, Clostridium and Staphylococcus*. The decreased β-glycosidase activity suggests the beneficial modification of intestinal microbiota composition by the CGAs-supplemented breads, particularly those enriched with the complex βCD-CGAs. This could be caused by the limited release of CGAs from the bread crumb in the upper gastrointestinal tract and thus the lower bioavailability and bioaccessibility in comparison with the CGAs from the FGCE and FβCD-CGAs diets. In consequence, the concentration of CGAs released in the colon from bread supplemented with the βCD-CGAs complex was higher that in turn had a positive impact on the growth of beneficial microflora. When the diets were supplemented with the preparations FGCE and FβCD-CGAs, CGAs were partially absorbed in the upper part of the gastrointestinal tract, even when they were included in the complex with β-CD [16, 24].

**Table 3:** The activity of some bacterial enzymes in the faeces of rats during the experiment

Enzyme	C	CF	FGCE	FβCD-CGAs	CB	BGCE	BβCD-CGAs	SEM	*P*
β-glucosidase									
Day 0	47.6	46.5^a^	48.2	46.5^a^	46.3	45.6^a^	45.2	1.632	0.074
Day 4	21.4	29.0	20.3	17.7	26.2	11.9	10.9	1.765	0.097
Day 8	18.4	21.4^a^	15.5	12.3	22.4^a^	8.0	5.3	1.362	0.081
Day 11	14.7	18.2	7.2^a^	7.1^a^	15.9	5.7	4.3	0.856	0.116
β-galactosidase									
Day 0	76.8^b^	76.8^b^	77.2	76.9^b^	76.4^a^	76.4^a^	74.1	0.885	0.095
Day 4	65.7	138.0	130.7	111.4	66.7	41.2	38.5	3.095	0.125
Day 8	56.1	127.3	119.2	97.8	61.0	25.5	23.4	3.497	0.041
Day 11	48.8	113.1	96.3	76.6	45.1	27.6	25.4	2.504	0.023
β-glucuronidase									
Day 0	38.1^c^	37.0^a,b^	36.8^a^	37.3^b^	39.5^c^	38.0	36.9^a^	2.100	0.072
Day 4	49.2	75.3	56.3	51.2	47.1	35.5	30.7	3.279	0.008
Day 8	20.8	69.9	37.2	22.9	29.5	14.7	10.1	2.086	0.075
Day 11	14.0	55.8	27.2	22.0	18.2	8.9^a^	8.8^a^	1.291	0.124

*SEM* Summed standard error; *n* = 56; μmol/h/g faeces; the same letter in one line means no significant differences (*P* > 0.05); for abbreviations of diets see the descriptions under Table 2

### Concentration of Volatile Short Chain Fatty Acids (SCFA) in the Rat Cecum Contents

Differences in the composition of the diets used in the study caused that the volatile fatty acids concentrations in the cecum contents were different (Table 4). The lowest concentration of putrefactive short chain fatty acids (PSCFA, the sum of iso-butyric, iso-valeric and valeric acids) was found in the groups fed the supplemented bread, particularly enriched with the BβCD-CGAs. In case of rats consuming the FGCE and FβCD-CGAs diets, the PSCFA concentration was also lower than in the rats fed the CF and C diets. The concentration of all analyzed short chain fatty acids (SCFA), including also acetic, propionic and butyric acids, produced by bifidogenic bacteria was statistically lower in the control groups compared to the groups fed the diets supplemented with CGAs. The positive effect of CGAs administered as inclusion complexes, observed as the high SCFA concentration, was also observed (*P* < 0.05). These data provide evidence of the beneficial modification of microbiota composition by the diets supplemented with phenolic acids from coffee, which was also observed by Rao et al. [23]. The inclusion of CGAs inside the molecules of β-cyclodextrin increased their bioavailability because of the limited interactions with the other components of the diet and bread [13, 17, 25].

**Table 4:** Concentration of volatile short chain fatty acids (SCFA) in the contents of the cecum in rats

Fatty acid	C	CF	FGCE	FβCD-CGAs	CB	BGCE	BβCD-CGAs	SEM	*P*
Acetic acid	32.4	38.2	41.0	43.4	38.8	45.7	46.6	1.973	0.003
Propionic acid	12.5	7.1	14.8	17.2	12.0	16.5	19.7	0.510	0.009
Iso-butyric acid	4.0^c,d^	3.9^c^	3.1^b^	3.0^b^	4.2^d^	2.8^a^	2.8^a^	0.127	0.043
Butyric acid	3.5	2.1	4.7	5.0	3.2	5.3^a^	5.4^a^	0.218	0.003
Iso-valeric acid	2.3^c^	2.6	2.1^c^	1.5^a^	1.7^b^	1.4^a,b^	1.2	0.090	0.034
Valeric acid	1.9	2.1	1.2	0.8^a^	0.8^a^	0.6^a^	0.5^a^	0.047	0.001
PSCFA	8.2	8.6	6.4	5.3	6.7	4.8	4.5	0.211	0.306
SCFA Total	56.6	56.0	66.9	70.9	60.7	72.3	76.2	2.635	0.004

*SEM* Summed standard error, *n* = 56; *PSCFA* Putrefactive volatile fatty acids (sum of iso-butyric, iso-valeric and valeric acids); μmol/g of cecum content; the same letter in one line means no significant differences (*P* > 0.05); for abbreviations of diets see the descriptions under Table 2

### Selected Indices Related to Metabolic Syndrome in Rats Blood Serum

The diet composition affected biochemical indices of rats blood serum (Table 5). Glucose concentrations in rats’ blood serum was the lowest in the FGCE and FβCD-CGAs diet groups, while the level of triglycerides was lower in the BGCE and BβCD-CGAs diet groups than in the other ones. The level of total cholesterol was relatively high in the CF and FβCD-CGAs as well as BβCD-CGAs diet groups, however the groups fed the diets with supplemented bread showed the highest levels of HDL (*P* < 0.05). Among the indices related to the metabolism of nitrogen containing compounds, the differences in uric acid concentrations in the blood serum were noted. The urea and creatinine levels were lower in the groups supplemented with the GCE (also added to bread) compared to the groups fed the C, CF and CB diets. The supplementation of diet with CGAs positively influenced the activities of aminotransferases that are indices of the liver status. The activities of aspartate and alanine aminotransferases were lower in the groups fed bread supplemented with both forms of CGAs.

**Table 5:** Selected indices associated with metabolic syndrome in rats blood serum

Index	C	CF	FGCE	FβCD-CGAs	CB	BGCE	BβCD-CGAs	SEM	*P*
Glucose (mmol/L)	11.5^a^	11.8^b^	10.8	11.1	11.5^a^	11.7^b^	11.7^b^	0.198	0.062
TG (mmol/L)	0.64^b^	0.67^c^	0.64^b^	0.61	0.66^c^	0.58^a^	0.57^a^	0.022	0.081
TC (mmol/L)	1.31	1.58^b^	1.34^a^	1.59^b^	1.33^a^	1.38	1.50	0.037	0.124
HDL (mmol/L)	0.53^a^	0.54^a^	0.59^b^	0.66	0.53^a^	0.58^b^	0.64	0.014	0.026
non-HDL (mmol/L)	0.78^a^	1.04	0.75	0.93	0.80^a^	0.80^a^	0.86	0.027	0.174
Uric acid (μmol/L)	41.3	36.6^a^	32.8	36.0	41.8	34.5	36.4^a^	1.256	0.113
Creatynine (μmol/L)	23.2^b,c^	23.1^b^	19.4	19.7^a^	23.4^c^	18.8	19.6^a^	0.986	0.076
Urea (mmol/L)	5.03	4.30	3.64	4.12^a,b^	4.17^c^	4.08^a^	4.15^b,c^	0.147	0.050
AST (U/L)	75.0^a^	82.7	77.9	80.6	75.1^a^	71.9	73.1	1.653	0.099
ALT (U/L)	25.3	27.1	21.4	24.0	21.8	20.1	20.8	0.610	0.111

*SEM* Summed standard error, *n* = 56; *TG* Triglycerides; *TC* Total cholesterol; *HDL-HDL* Cholesterol; *AST* Aspartate aminotransferase; *ALT* Alanine aminotransferase; the same letter in one line means no significant differences (*P* > 0.05); for abbreviations of diets see the descriptions under Table 2

### Antioxidant Indices in the Internal Organs and Blood Serum of Rats

The diets of different composition influenced the indices related to the antioxidant status in rats, which is characterized by the antioxidant capacity of hydrophilic and lipophilic (ACW and ACL, respectively) fractions of blood serum (Table 6). The values of ACW and ACL were the highest in rats fed the FβCD-CGAs and BβCD-CGAs diets that may be caused by the increased bioaccessibility of CGAs, resulting from the reduced interaction with proteins from the diet [25]. The groups differed in the concentrations of substances reacting with thiobarbituric acid (TBARS) in internal organs. The content of TBARS in the heart muscle was the lowest in the BGCE diet fed group, and in the kidney it was the lowest in the FGCE diet group. In these two groups, significant amounts of CGAs could be absorbed in the small intestine, getting rapidly into the bloodstream and causing the observed health-promoting activities [26]. In the groups fed the diets containing extracts from green coffee beans, a higher ratio of reduced to oxidized glutathione (GSH/GSSG) was observed in rats’ liver, demonstrating an increase in the antioxidant capacity of this organ. The results indicated that the increase in the GSH level was not caused only by caffeine consumption that was demonstrated previously by Cavin et al. [27]. The high antioxidant indices of blood serum and different organs in animals fed CGAs in the form of inclusion complexes provided evidence of the beneficial effect of microencapsulation on the maintenance of antioxidant activity, being a result of the high bioaccessibility of CGAs. This observation may explain the increased antitumor activity of CGAs derivatives protected by microencapsulation [28].

**Table 6:** Antioxidant indices in the internal organs and blood serum in rats

Index	C	CF	FGCE	FβCD-CGAs	CB	BGCE	BβCD-CGAs	SEM	*P*
Serum									
ACW (μg/mL)	1.59^a^	1.52	1.75	1.97	1.57^a^	1.70	2.09	0.075	0.051
ACL (μg/mL)	1.51^a,b^	1.50^a^	2.19	4.97	1.54^b^	1.85	4.30	0.262	0.101
Heart									
Weight (g/100 g BW)	0.255a	0.268^b^	0.255a	0.265^b^	0.255^a^	0.249	0.242	0.003	0.176
TBARS	781	827^a^	794	826^a^	801	705	789	27.45	0.126
Kidney									
Weight (g/100 g BW)	0.582^b^	0.545^a^	0.570^c^	0.547^a^	0.586^b^	0.564^c^	0.606	0.007	0.023
TBARS	1186	1248	1104	1138	1154	1125	1167	12.38	0.192
Liver									
Weight (g/100 g BW)	2.78^b,c^	2.81^c^	2.62	2.78^b,c^	2.76^a,b^	2.70	2.75^a^	0.025	0.117
TBARS	708	770	722	672	704	756	746	10.63	0.105
GSH (μmol/g)	16.8^a^	10.0	19.5^b^	12.7	16.9^a^	19.9^c^	19.6^b,c^	0.478	0.081
GSSG (μmol/g)	5.82^a^	5.46	2.91	5.84^a^	5.96	6.33	5.57	0.199	0.107
GSH/GSSG	2.89^a^	1.83	6.70	2.17	2.84^a^	3.14	3.52	0.127	0.031

*SEM* Summed standard error, *n* = 56; *TBARS* Content of substances reacting with thiobarbituric acid, ng/g tissue; *ACW/ACL* The antioxidant capacity derived from hydrophilic/lipophilic antioxidants; the same letter in one line means no significant differences (*P* > 0.05); for abbreviations of diets see the descriptions under Table 2

## Conclusions

It is known that chlorogenic acids have the antioxidant potential. However, the potential of inclusion complexes of chlorogenic acids and β-cyclodextrin and the effect of fermentation and baking on chlorogenic acids added to bread, also after microencapsulation, have not been studied earlier. The results of our study clearly showed that chlorogenic acids added to bread positively affected enzymatic activities of intestinal microbiota, because of the reduced absorption in the small intestine and increased passage to the colon. The appearance of CGAs in the colon decreased the activity of β-glycosidases, reduced the concentrations of urea and putrefactive short chain fatty acids, and lowered the pH of the intestinal contents. The CGAs preparations added directly to the diet were highly absorbed in the small intestine and contributed to the improved antioxidant status in the liver and kidneys, decreased glucose concentration and increased content of HDL in the blood serum. The high GSH/GSSG ratio in the liver and high concentration of the antioxidants in blood serum was observed as a result of the consumption of diets containing chlorogenic acids in the form of inclusion complexes with β-cyclodextrin. This indicates that microencapsulation allows to increase the bioaccessibility of chlorogenic acids, due to the limited interactions with other components of the diet. This is the first report on the antioxidant activity of chlorogenic acids complexed with β-dextrin, which makes them promising candidates for prevention of the oxidative stress and associated metabolic disorders. The results presented in this work may pave the way for further studies on the influence of chlorogenic acids dosage and the type of food being their carrier, on mitigation of the oxidative stress symptoms.

## References

[1] Biesalski HK, Dragsted LO, Elmadfa I, Grossklaus R, Müller M, Schrenk D, Walter P, Weber P (2009). Bioactive compounds: definition and assessment of activity. Nutrition.

[2] Dziki D, Gawlik-Dziki U, Pecio Ł, Różyło R, Świeca M, Krzykowski A, Rudy S (2015). Ground green coffee beans as a functional food supplement - preliminary study. LWT-Food Sci Technol.

[3] Budryn G, Rachwał-Rosiak D (2013). Interactions of hydroxycinnamic acids with proteins and their technological and nutritional implications. Food Rev Int.

[4] Budryn G, Zaczyńska D, Oracz J (2016). Effect of addition of free and nanoencapsulated chlorogenic acids on aroma of different food products. LWT-Food Sci Technol.

[5] Budryn G, Nebesny E, Rachwał-Rosiak D, Pałecz B, Hodurek P, Miśkiewicz K, Oracz J, Żyżelewicz D (2014). Inclusion complexes of β-cyclodextrin with chlorogenic acids from crude and purified aqueous extracts from green Robusta coffee beans (*Caffea canephora* L.). Food Res Int.

[6] Jeszka-Skowron M, Sentkowska A, Pyrzyńska K, Paz De Peńa M (2016). Chlorogenic acids, caffeine content and antioxidant properties. Eur Food Res Technol.

[7] Satake T, Kamiya K, An Y, Oishi T, Yamamoto J (2007). The anti-thrombotic active constituents from *Centella asiatica*. Biol Pharm Bull.

[8] Lee KW, Im JY, Woo JM, Grosso H, Kim YS, Cristovao AC, Sonsalla PK, Schuster DS, Jalbut MM, Fernandez JR, Voronkov M, Junn E, Braithwaite SP, Stock JB, Mouradian MM (2013). Neuroprotective and anti-inflammatory properties of a coffee component in the MPTP model of Parkinson's disease. Neurotherapeutics.

[9] Shi H, Dong L, Dang X, Liu Y, Jiang J, Wang Y, Lu X, Guo X (2013). Effect of chlorogenic acid on LPS-induced proinflammatory signaling in hepatic stellate cells. Inflamm Res.

[10] Silva BA, Ferreres F, Malva JO, Dias ACP (2005). Phytochemical and antioxidant characterization of *Hypericum perforatum* alcoholic extracts. Food Chem.

[11] Lee KW, Lee HJ (2006). The roles of polyphenols in cancer chemoprevention. BioFactor.

[12] Cheng JC, Dai F, Zhou B, Yang L, Liu ZL (2007). Antioxidant activity of hydroxycinnamic acid derivatives in human low density lipoprotein: mechanism and structure-activity relationship. Food Chem.

[13] Abrahão SA, Pereira RG, de Sousa RV, Lima AR, Crema GP, Barros BS (2013). Influence of coffee brew in metabolic syndrome and type 2 diabetes. Plant Foods Hum Nutr.

[14] Glei M, Kirmse A, Habermann N, Persin C, Pool-Zobel BL (2006). Bread enriched with green coffee extract has chemoprotective and antigenotoxic activities in human cells. Nutr Cancer.

[15] Szejtli J, Szente L (2005). Elimination of bitter, disgusting tastes of drugs and foods by cyclodextrins. Eur J Pharm Biopharm.

[16] Paramera EI, Konteles SJ, Karathanos VT (2011). Stability and release properties of curcumin encapsulated in *Saccharomyces cerevisiae*, β-cyclodextrin and modified starch. Food Chem.

[17] Budryn G, Nebesny E, Rachwał D (2014). Pepsin digestibility and antioxidant activity of egg white protein in model systems with green coffee extract. Int J Food Prop.

[18] Budryn G, Pałecz B, Rachwał-Rosiak D, Oracz J, Zaczyńska D, Belica S, Navarro-González I, Meseguer JM, Pérez-Sánchez H (2015). Effect of inclusion of hydroxycinnamic and chlorogenic acids from green coffee bean in β-cyclodextrin on their interactions with whey, egg white and soy protein isolates. Food Chem.

[19] Żyżelewicz D, Zakłos-Szyda M, Juśkiewicz J, Bojczuk M, Oracz J, Budryn G, Miśkiewicz K, Krysiak W, Zduńczyk Z, Jurgoński A (2016). Cocoa bean (*Theobroma cacao* L.) phenolic extracts as PTP1B inhibitors, hepatic HepG2 and pancreatic β-TC3 cell cytoprotective agents and their influence on oxidative stress in rats. Food Res Int.

[20] Choi BK, Park SB, Lee DR, Lee HJ, Jin YY, Seung HY, Sub JW (2016). Green coffee bean extract improves obesity by decreasing body fat in high-fat diet-induced obese mice. Asian Pac J Trop Med.

[21] Kobayashi-Hattori K, Mogi A, Matsumoto Y, Takita T (2005). Effect of caffeine on the body fat and lipid metabolism of rats fed on a high-fat diet. Biosci Biotechnol Biochem.

[22] Kumao T, Fujii S (2006). Mannooligosaccharides blended coffee beverage intake increases the fat level in feces. J Health Sci.

[23] Rao CV, Chou D, Simi B, Ku H, Reddy BS (1998). Prevention of colonic aberrant crypt foci and modulation of large bowel microbial activity by dietary coffee fiber, inulin and pectin. Carcinogenesis.

[24] Guy PA, Renouf M, Barron D, Cavin C, Dionisi F, Kochhar S, Rezzi S, Williamson G, Steiling H (2009). Quantitative analysis of plasma caffeic and ferulic acid equivalents by liquid chromatography tandem mass spectrometry. J Chromatogr B.

[25] Budryn G, Zaczyńska D, Rachwał-Rosiak D (2016). Effect of green coffee polyphenols on properties of protein hydrolysates in model systems. J Food Process Preserv.

[26] Renouf M, Guy PA, Marmet C, Fraering AL, Longet K, Moulin J, Enslen M, Barron D, Dionisi F, Cavin C, Williamson G, Steiling H (2010). Measurement of caffeic and ferulic acid equivalents in plasma after coffee consumption: small intestine and colon are key sites for coffee metabolism. Mol Nutr Food Res.

[27] Cavin C, Marin-Kuan M, Langouët S, Bezençon C, Guignard G, Verguet C, Piguet D, Holzhäuser D, Cornaz R, Schilter B (2008). Induction of Nrf2-mediated cellular defenses and alteration of phase I activities as mechanisms of chemoprotective effects of coffee in the liver. Food Chem Toxicol.

[28] Yu H, Huang Q (2010). Enhanced *in vitro* anti-cancer activity of curcumin encapsulated in hydrophobically modified starch. Food Chem.

